# CTLA-4 Synergizes With PD1/PD-L1 in the Inhibitory Tumor Microenvironment of Intrahepatic Cholangiocarcinoma

**DOI:** 10.3389/fimmu.2021.705378

**Published:** 2021-08-30

**Authors:** Xiao-Jun Guo, Jia-Cheng Lu, Hai-Ying Zeng, Rong Zhou, Qi-Man Sun, Guo-Huan Yang, Yan-Zi Pei, Xian-Long Meng, Ying-Hao Shen, Peng-Fei Zhang, Jia-Bin Cai, Pei-Xin Huang, Ai-Wu Ke, Ying-Hong Shi, Jian Zhou, Jia Fan, Yi Chen, Liu-Xiao Yang, Guo-Ming Shi, Xiao-Yong Huang

**Affiliations:** ^1^Department of Liver Surgery and Transplantation, Zhongshan Hospital, Fudan University, Shanghai, China; ^2^Liver Cancer Institute, Fudan University, Shanghai, China; ^3^Key Laboratory of Carcinogenesis and Cancer Invasion, Ministry of Education of the People’s Republic of China, Shanghai, China; ^4^Department of Pathology, Zhongshan Hospital, Fudan University, Shanghai, China; ^5^Department of Transfusion, Zhongshan Hospital, Fudan University, Shanghai, China; ^6^Department of Medical Oncology, Zhongshan Hospital, Fudan University, Shanghai, China; ^7^Department of Critical Care Medicine, Zhongshan Hospital, Fudan University, Shanghai, China

**Keywords:** intrahepatic cholangiocarcinoma, cytotoxic T-lymphocyte-associated antigen-4, programmed death ligand-1, prognosis, hepatolithiasis

## Abstract

Intrahepatic cholangiocarcinoma (ICC) is highly invasive and carries high mortality due to limited therapeutic strategies. In other solid tumors, immune checkpoint inhibitors (ICIs) target cytotoxic T lymphocyte-associated antigen 4 (CTLA-4) and programmed death 1 (PD1), and the PD1 ligand PD-L1 has revolutionized treatment and improved outcomes. However, the relationship and clinical significance of CTLA-4 and PD-L1 expression in ICC remains to be addressed. Deciphering CTLA-4 and PD-L1 interactions in ICC enable targeted therapy for this disease. In this study, immunohistochemistry (IHC) was used to detect and quantify CTLA-4, forkhead box protein P3 (FOXP3), and PD-L1 in samples from 290 patients with ICC. The prognostic capabilities of CTLA-4, FOXP3, and PD-L1 expression in ICC were investigated with the Kaplan–Meier method. Independent risk factors related to ICC survival and recurrence were assessed by the Cox proportional hazards models. Here, we identified that CTLA-4^+^ lymphocyte density was elevated in ICC tumors compared with peritumoral hepatic tissues (*P* <.001), and patients with a high density of CTLA-4^+^ tumor-infiltrating lymphocytes (TILs^CTLA-4 High^) showed a reduced overall survival (OS) rate and increased cumulative recurrence rate compared with patients with TILs^CTLA-4 Low^ (*P* <.001 and *P* = .024, respectively). Similarly, patients with high FOXP3^+^ TILs (TILs^FOXP3 High^) had poorer prognoses than patients with low FOXP3^+^ TILs (*P* = .021, *P* = .034, respectively), and the density of CTLA-4^+^ TILs was positively correlated with FOXP3^+^ TILs (Pearson *r* = .31, *P* <.001). Furthermore, patients with high PD-L1 expression in tumors (Tumor^PD-L1 High^) and/or TILs^CTLA-4 High^ presented worse OS and a higher recurrence rate than patients with TILs^CTLA-4 Low^Tumor^PD-L1 Low^. Moreover, multiple tumors, lymph node metastasis, and high Tumor^PD-L1^/TILs^CTLA-4^ were independent risk factors of cumulative recurrence and OS for patients after ICC tumor resection. Furthermore, among ICC patients, those with hepatolithiasis had a higher expression of CTLA-4 and worse OS compared with patients with HBV infection or undefined risk factors *(P* = .018). In conclusion, CTLA-4 is increased in TILs in ICC and has an expression profile distinct from PD1/PD-L1. Tumor^PD-L1^/TILs^CTLA-4^ is a predictive factor of OS and ICC recurrence, suggesting that combined therapy targeting PD1/PD-L1 and CTLA-4 may be useful in treating patients with ICC.

## Introduction

Evasion of immune destruction is a hallmark of cancer and results in immune tolerance (1). Immune tolerance can be mediated through multiple pathways, including the immune checkpoint receptors cytotoxic T-lymphocyte-associated antigen 4 (CTLA-4) and programmed cell death protein 1 (PD1) (2). A costimulatory signal exerted by CD28:B7 binding is necessary for T cell maturation; CTLA-4 belongs to the CD28 family of immunoglobulins and competitively binds to B7 to produce inhibitory signals that counteract stimulatory signals from CD28:B7 and TCR: MHC binding (3). CTLA-4: B7-1/2 (CD80/B7-1 and CD86/B7-2) binding suppresses several signaling cascades in T cells, including differentiation, proliferation, and survival through inhibits IL-2 accumulation and cell cycle progression etc. (4, 5). CTLA-4 maintains peripheral tolerance by enhancing regulatory T cell (Treg) functions; thus, undirected control of the effector T cells (6) and overexpression of CTLA-4 in tumor samples implicates poor prognosis in patients with melanoma (7). Because CTLA-4 inhibition results in increased activation of the immune system, Ipilimumab, an inhibitor of CTLA-4, was approved for the treatment of advanced or unresectable melanoma (8).

PD1 regulates the activation of T cells by binding to programmed death-ligand 1/2 (PD-L1/2). Activation of the PD1/PD-L1/L2 pathway inhibits T cell proliferation and secretion of interferon-gamma (IFN-γ), tumor necrosis factor-alpha (TNF-α), and IL-2, sustaining the immune inhibitory state of the tumor microenvironment (9). Clinical evidence supports aberrant PD-L1 expression in tumor cells, which aids in their escape from T cell immune attack in non-small-cell lung cancer (NSCLC), renal cell carcinoma, Hodgkin’s lymphoma, hepatocellular carcinoma (HCC), and intrahepatic cholangiocarcinoma (ICC) (10). Based on these findings, immune checkpoint inhibitors (ICIs) targeting PD1/PD-L1 are approved or being evaluated as a malignant tumor treatment in various tumors (11).

Although CTLA-4 and PD1/PD-L1 exert similar negative effects on T cell activity, their timing and mechanisms differ. CTLA-4 acts in the initial stage of the immune response, typically in lymph nodes, and the PD1/PD-L1 pathway regulates previously activated T cells at later stages primarily in peripheral tissues (12). Recent evidence reveals interactions between PD1/PD-L1 and CTLA-4 signals. For example, the tumor cell glycolytic rate is depressed by anti-PD-L1 therapy, and patients with low glucometabolic levels in tumors may benefit from CTLA-4 blockade (13, 14). ^A^ combination of anti-CTLA-4 and anti-PD1/PD-L1 therapies may have an additive or synergistic effect in the treatment of advanced malignancies. Preliminary results of a clinical study report that the combination of the anti-CTLA-4 antibody ipilimumab and anti-PD1 antibody nivolumab elevated the objective response rate (ORR) and the progression-free survival of patients with BRAF^WT^ metastatic or unresectable melanoma (15).

ICC accounts for 10%–15% of primary liver cancer cases, but its incidence has rapidly increased worldwide, and major ICC risk factors are hepatitis virus B (HBV) and C (HCV) infection along with hepatolithiasis (16, 17). ICC has a poor prognosis owing to local invasion and distal metastasis at first diagnosis (18). First-line therapy for ICC is gemcitabine-based chemotherapy as it is for other advanced biliary tract tumors, but even with treatment, the median overall survival (OS) is 11.7 months (19). Our previous study revealed elevated PD1/PD-L1 signals in tumor samples and distinct profiles of PD-1/PD-L1 in ICC patients with different risk factors (20), and the PD1 inhibitor Toripalimab, in combination with GEMOX (oxaliplatin and gemcitabine) chemotherapy and Lenvatinib, showed an ORR of 80% (24/30) and a 93.3% (28/30) disease control rate in treating advanced ICC (21). On the other hand, CTLA-4 expression and its relationship with tumor-infiltrating Tregs has not been characterized in ICC, and little is known about CTLA-4 and PD1/PD-L1 expression and interaction in ICC. This information could guide both diagnosis and treatment. To address this knowledge gap, we investigated the expression and interaction of CTLA-4 and PD1/PD-L1 in ICC and assessed their value as prognostic indicators in ICC.

## Materials and Methods

### Patients and Clinical Samples

Study participants consisted of 290 patients with ICC who underwent curative resection between May 2002 and December 2011 at Zhongshan Hospital, Fudan University. Enrolled patients met the following criteria: (1) pathologically confirmed ICC; (2) ≥3 months of disease-free survival (DFS) after resection; (3) had not undergone antitumor treatment before surgery; and (4) had complete medical records and follow-up data available. Patients were stratified by a tumor-node-metastases (TNM) stage system according to the American Joint Committee on Cancer (AJCC) 8^th^ edition (22). The histological grade of ICC was based on World Health Organization criteria (23). Tumor samples and adjacent liver tissue samples were collected, formalin-fixed, and paraffin-embedded. The last follow-up was on April 30, 2016. The study was approved by the institutional review board of Zhongshan Hospital (Y2017-130), and all related procedures conformed to the Declaration of Helsinki.

### Tissue Microarrays and Immunohistochemistry

We previously described methods for the construction of tissue microarrays (TMAs) and immunohistochemistry (IHC) (24). Briefly, antihuman rabbit monoclonal antibodies for FOXP3 (1:50; #98377S, CST, Massachusetts, USA) and antihuman mouse monoclonal antibodies for CTLA-4 (1:100; #ab19792, Abcam, Cambridge, UK) were used as primary antibodies to detect the expression of FOXP3 and CTLA-4. An automated digital pathological slice scanner, KF-PRO-120 (KONFOONG Biotech International Co. Ltd., Ningbo, China), was used to scan images of IHC slides, and slides were photographed by digital slices view software K-Viewer (KONFOONG). IHC for PD-L1 was performed as described (20).

### Evaluation of CTLA-4 and FOXP3 Expression

The previous study in extrahepatic bile duct cancer revealed CTLA-4 expressed on both tumor cells and TILs (25); here, we found that CTLA-4 positively stained both tumor cells and interstitial cells as well, and CTLA-4^+^ TILs were distinguished by their topographic localization, cell nucleus volume, and other morphological characteristics. Two independent pathologists evaluated the expression of CTLA-4, and FOXP3 as a marker of Tregs was also evaluated to reveal the relationship between CTLA-4 and tumor-infiltrating Tregs (26). Lymphocytes with positive staining for CTLA-4 and FOXP3 were manually counted in five high-power fields that were randomly selected under 200× magnification for each TMA core, and the mean density (the number of positively stained cells per field) was determined to represent the expression level for each patient. Positive expression of CTLA-4 in tumor cells was scored 0–5 (0, <5% of the tissue section; 1, 5%–40%; 2, 40%–75%; 3, 75%–85%; 4, 85%–95%; 5, ≥95%). The median number of CTLA-4 and FOXP3-positive infiltrating lymphocytes was defined as the cutoff value for high or low expression levels. By calculating the Youden index, patients with CTLA-4 expression on tumor cells were divided into high (score >2) and low (score ≤2) score subgroups. PD-L1 expression was evaluated as described (20).

### Statistical Analyses

Statistical analyses were performed with SPSS 25.0 (Chicago, IL, USA), R (version 4.0.2, R foundation for statistical, Vienna, Austria), and GraphPad Prism 8 software (La Jolla, CA, USA). Values are presented as median (range) or mean ± standard deviation (SD). Paired Student’s *t*-test, χ^2^ tests, one-factor analysis of variance (one-way ANOVA), Pearson correlation analysis, Spearman rank correlation analysis, and the Wilcoxon rank-sum test were used to compare differences between groups. The Kaplan–Meier method was used to construct the survival and recurrence curves. Cox proportional hazards model analysis was used to analyze the correlation between variables and ICC patient prognosis. Statistical tests were two-tailed, and P-values <.05 were considered significant.

## Results

### Expression and Prognostic Implication of CTLA-4 in ICC

**Figure 1:**
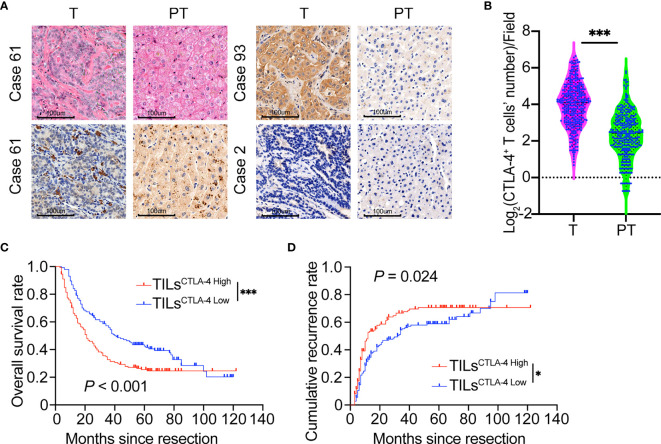
Prognostic implications of CTLA-4 expression in ICC tumors versus paired adjacent normal liver tissues. **(A)** Representative H&E staining of CTLA-4 in ICC tumor and paired adjacent normal liver tissues (Case 61, CTLA-4 positively stained on tumor-infiltrating lymphocytes; Case 93, CTLA-4 positively stained on ICC tumor cells; Case 2, Negative staining on tumor cells or lymphocytes of CTLA-4; T, Tumor; PT, Paired adjacent normal liver tissues). Magnification 200x. **(B)** Density of CTLA-4^+^ infiltrating lymphocytes was higher in ICC tissues than paired adjacent normal liver tissues in the whole ICC cohort (*P* <0.001, paired Student’s *t-*test). **(C, D)** The Kaplan–Meier curve of OS and cumulative recurrence shows that patients with TILs^CTLA-4 High^ were associated with worse OS and a higher cumulative recurrence rate compared with patients with TILs^CTLA-4 Low^. *P < 0.05 and ***P < 0.001.

The distribution of positive CTLA-4 expression in ICC was highly heterogeneous (**Figure 1A**). CTLA-4 positive staining is mainly localized in lymphocytes, the tumor cell membrane, and the hepatocyte cytoplasm in adjacent liver tissues. CTLA-4 is transferred from intracellular vesicles to the cell surface after environmental stimulation and plays a role in sustaining the inhibitory tumor environment (27, 28). Here, we investigated the expression of CTLA-4 in the membrane of T cells and tumor cells. The density of positively stained CTLA-4^+^-infiltrating lymphocytes in the tumor tissue was 22.0 ± 19.1/field, which was significantly higher than in para-tumor hepatic tissue (7.5 ± 7.8/field, *P* <.001, **Figure 1B**). The different expression levels of CTLA-4 in tumor cells are presented in **Supplementary Material, Figure S1**.

By final follow-up, 177 patients experienced relapsed disease, and median DFS was 14 months (range, 3–122 months). Postoperative 2-, 5-, and 10-year recurrence rates were 53.8%, 64.5%, and 79.4%, respectively. Two hundred patients died, and the median OS was 24.5 months (range, 3–122 months). The 2-, 5-, and 10-year postoperative survival rates were 55.5%, 34.0%, and 19.1%, respectively. Analysis of the relationship between CTLA-4 expression and patient prognosis revealed that patients with low CTLA-4 density in TILs (TILs^CTLA-4 Low^) had a much longer OS (*P* <.001) and a lower recurrence rate (*P* = .024) compared with patients with high density (TILs^CTLA-4 High^) (**Figures 1C, D**). However, the density of CTLA-4^+^ lymphocytes in adjacent hepatic tissues was not related to patient prognosis in terms of OS (*P* = .111) or recurrence rates (*P* = .057) (**Supplementary Material, Figures S2A, B**). We also analyzed the prognostic role of CTLA-4 expression in tumor cells (Tumor^CTLA-4^) in patients with ICC. No statistical difference in OS (*P* = .402) or recurrence rate (*P* = .080) was observed between Tumor^CTLA-4 High^ and Tumor^CTLA-4 Low^ subgroups (**Supplementary Material, Figures S2C, D**).

Moreover, a higher density of TILs^CTLA-4^ was related to malignant characteristics in ICC, including a higher level of preoperative serum CA19-9 (*P* = .003), larger tumor size (*P* = .014), lymph node metastasis (*P* = .019), and high TNM stage (*P* = .036). Other parameters were not related to CTLA-4 expression; detailed information is listed in **Table 1**.

**Table 1:** Correlation between CTLA-4 and clinicopathological features in 290 patients with ICC.

Features	TILs^CTLA-4^		
	Low	High	*P* value
Age (y)			
<58	72	67	.484
≥58	72	79	
Sex			
Female	56	58	.884
Male	88	88	
Hepatolithiasis			
Negative	138	136	.317
Positive	6	10	
HBV infection			
Negative	40	37	.639
Positive	104	109	
Liver cirrhosis			
Negative	106	108	.944
Positive	38	38	
ALT(U/L)			
<75	136	135	.496
≥75	8	11	
AFP (ng/mL)			
<20	127	128	.891
≥20	17	18	
CA19-9(U/L)			
<37	89	65	.003
≥37	55	81	
Tumor size(cm)			
≤5	78	58	.014
>5	66	88	
Tumor number			
Single	116	110	.284
Multiple	28	36	
Tumor differentiation			
I/II	92	85	.322
III/IV	52	61	
Lymph node metastasis			
Negative	128	115	.019
Positive	16	31	
Nerve invasion			
Negative	137	136	
Positive	7	10	.471
Microvascular invasion			
Negative	131	122	
Positive	13	24	.059
TNM stage			
I/II	121	108	
III	23	38	.036

HBV, hepatitis B virus; ALT, Alanine aminotransferase; AFP, alpha-fetoprotein; CA19-9, Carbohydrate antigen 19-9; TILs^CTLA-4^, the density of CTLA-4^+^ TILs.

### Expression Pattern of FOXP3 in ICC

**Figure 2:**
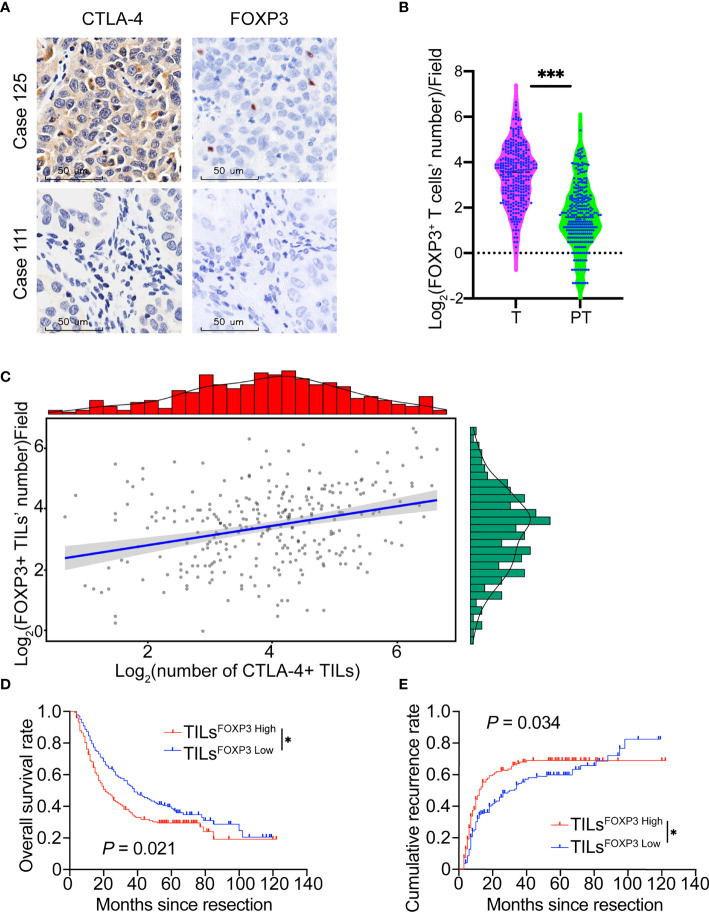
FOXP3 expression and relationship with CTLA-4 in patients with ICC. **(A)** Representative staining of CTLA-4 and FOXP3 in ICC tumor samples (Case 125: patient with a high density of CTLA-4^+^ TILs and FOXP3^+^ TILs; Case 111: patient with low density of CTLA-4^+^ TILs and FOXP3^+^ TILs). Magnification 400x. **(B)** Density of FOXP3^+^ infiltrating lymphocytes was higher in ICC tissues than paired adjacent normal liver tissues in the whole ICC cohort. **(C)** Positive correlation between the density of FOXP3^+^ TILs and CTLA-4^+^ TILs. **(D, E)** The Kaplan-Meier curve of OS and cumulative recurrence shows that patients with TILs^FOXP3 High^ were associated with worse OS and a higher cumulative recurrence rate compared with patients with TILs^FOXP3 Low^. *P < 0.05 and ***P < 0.001.

Because Treg cells maintain an inhibitory immune state in malignancies (29) and CTLA-4 inhibition could reduce Treg-mediated suppression of T cell responses (30), we further investigated FOXP3 expression and its relationship with CTLA-4 in patients with ICC. FOXP3 exhibited nuclear localization in lymphocytes (**Figure 2A**). The density of FOXP3^+^ TILs (TILs^FOXP3^) in ICC tumor samples was 15.7 ± 14.8/field, which is significantly higher than that in para-tumor liver tissues (4.8 ± 5.3/field, *P* <.001, **Figure 2B**) and lower than CTLA-4 (*P* <.001, **Figure S3A**). Pearson correlation analysis revealed a positive relationship between the density of CTLA-4^+^ TILs and FOXP3^+^ TILs (*r* = .31, *P* <.001, **Figure 2C**).

We also evaluated the prognostic potential of FOXP3 expression in ICC. Similar to studies in gastric and lymph node-positive breast cancer (31, 32), ICC patients with a high density of FOXP3^+^ TILs showed poor prognosis in terms of shorter OS (*P* = .021) and higher recurrence rates (*P* = .034, **Figures 2D, E**).

### Interaction Between CTLA-4/PD-L1 and Prognostic Implication in ICC

PD-1/PD-L1 and CTLA-4 had distinct modes of inhibitory T responses. Our previous studies (20) show that patients with high PD-L1 expression in ICC tumor cells (Tumor^PD-L1 High^) had a shorter OS and higher recurrence rates than patients with low tumor PD-L1.

**Figure 3:**
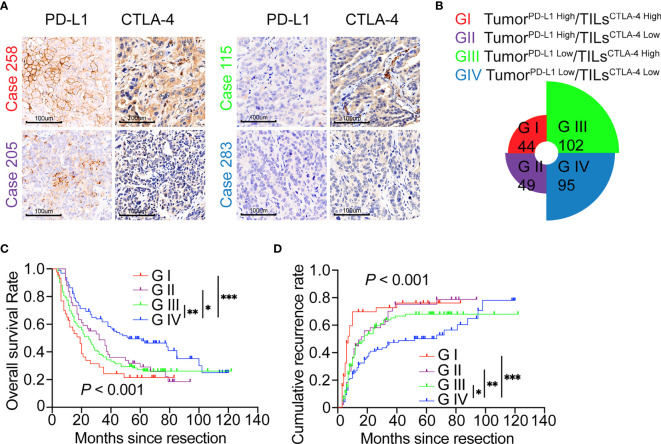
ICC classification and combined prognostic implications of PD-L1 and CTLA-4 expression. **(A, B)** Classification of patients with ICC according to PD-L1 expression in tumor cells and density of CTLA-4^+^ TILs along with representative staining pictures for each subgroup. Magnification 200x. **(C)** The Kaplan–Meier curve of OS shows that patients with Tumor^PD-L1 High^ or TILs^CTLA-4 High^ (GI/GII/GIII) are associated with worse OS compared with patients with Tumor^PD-L1 Low^ plus TILs^CTLA-4 Low^ (GIV). **(D)** The Kaplan–Meier curve of cumulative recurrence shows that patients with Tumor^PD-L1 High^ or TILs^CTLA-4 High^ (GI/GII/GIII) are associated with higher cumulative recurrence rates compared with patients with Tumor^PD-L1 Low^ plus TILs^CTLA-4 Low^ (GIV). *P < 0.05, **P < 0.01, and ***P < 0.001.

Here, in the whole cohort, Spearman rank correlation analysis revealed no correlation between the expression of TILs^CTLA-4^ and Tumor^PD-L1^ (*r* = .015, *P* = .802), suggesting that the expression of TILs^CTLA-4^ and Tumor^PD-L1^ in ICC was relatively independent. We next divided the cohort into four subgroups according to Tumor^PD-L1^ and TILs^CTLA-4^ expression (G I refers to Tumor^PD-L1 High^ and TILs^CTLA-4 High^ patients; G II refers to Tumor^PD-L1 High^ and TILs^CTLA-4 Low^ patients; G III refers to Tumor^PD-L1 Low^ and TILs^CTLA-4 High^ patients; G IV refers to Tumor^PD-L1 Low^ and TILs^CTLA-4 Low^ patients). Representative pictures are presented in **Figure 3A**. Commonly hyperactivated PD-L1 and CTLA-4 expression (G I) was observed in 44 patients, overexpression of PD-L1 alone (G II) in 49 patients, and overexpression of CTLA-4 alone (G III) in 102 patients. Low expression of PD-L1 and CTLA-4(G IV) was observed in 95 patients (**Figure 3B**).

Given the different activated states of PD-L1 and CTLA-4 pathways in tumor tissues, we further investigated the synthesized effect of Tumor^PD-L1^ and TILs^CTLA-4^ expression on prognosis in patients with ICC. Survival analysis showed that patients with Tumor^PD-L1 High^ and/or TILs^CTLA-4 High^ subgroups (G I, II, and III) had poorer prognoses in terms of shorter OS (G I *vs*. G IV, *P* <.001; G II *vs*. G IV, *P* = .017; G III *vs*. G IV, *P* = .001) and higher recurrence rates (G I *vs*. G IV, *P* <.001; G II *vs*. G IV, *P* = .008; G III *vs*. G IV, *P* = .046, log-rank test) compared with patients with Tumor^PD-L1 Low^ TILs^CTLA-4 Low^ (**Figures 3C, D**).

As for the FOXP3 expression level in the four groups’ patients, Tumor^PD-L1 High^/TILs^CTLA-4 Low^ patients have a higher level of Tregs compared with Tumor^PD-L1 Low^/TILs^CTLA-4 High^ patients (*P* <.001, **Figure S3B**), and integrally, Tumor^PD-L1 High^ patients are prone to have more Tregs infiltrating into the ICC tumor than Tumor^PD-L1 Low^ patients (*P* <.001, **Figure S3C**).

Cox regression analysis showed that clinicopathological characters, including tumor size and number, lymph node metastases, nerve invasion, TILs^FOXP3^, TILs^CTLA-4^, Tumor^PD-L1^, and Tumor^PD-L1^/TILs^CTLA-4^ were related to OS and recurrence rate of ICC. Hepatolithiasis was only related to patients’ survival (**Table 2**). Individual clinicopathological features that showed significance in univariate analysis, including tumor^PD-L1^ and TILs^CTLA-4^, were adopted as covariates in a multivariate Cox proportional hazards model (**Supplementary Material, Table S1**), and then combined variables of tumor^PD-L1^/TILs^CTLA-4^ were further analyzed (**Table 2**). Multiple tumors, lymph node metastasis, and tumor^PD-L1 High^ were determined as independent risk factors of cumulative recurrence for patients with ICC, and hepatolithiasis, large tumor, multiple tumors, lymph node metastasis, nerve invasion, and high TILs^CTLA-4^ were independent risk factors of OS. Interestingly, tumor^PD-L1^/TILs^CTLA-4^ was an independent risk factor of patient prognosis for ICC in terms of recurrence and OS (**Table 2**).

**Table 2:** Univariate and multivariate analyses of characteristics associated with prognosis in 290 patients with ICC.

Characteristics	Univariate analysis				Multivariate analysis			
	Cumulative recurrence		OS		Cumulative recurrence		OS	
	HR (95%CI)	*P* value	HR (95%CI)	*P* value	HR (95%CI)	*P* value	HR (95%CI)	*P* value
Age, years (>58 *vs* ≤58)	0.823 (0.612-1.106)	.196	0.894 (0.677-1.18)	.429	NA	NA	NA	NA
Sex (male *vs* female)	1.174 (0.865-1.594)	.302	1.187 (0.89-1.583)	.239	NA	NA	NA	NA
Hepatolithiasis (positive *vs* negative)	1.923 (0.979-3.777)	.058	3.932 (2.303-6.713)	<.001	NA	NA	4.326 (2.497-7.494)	<.001
HBV infection (positive *vs* negative)	1 (0.715-1.399)	.999	0.885 (0.649-1.205)	.436	NA	NA	NA	NA
Liver cirrhosis (positive *vs* negative)	1.173 (0.839-1.640)	.350	1.224 (0.896-1.670)	.204	NA	NA	NA	NA
Tumor differentiation (III/IV *vs* I/II)	1.283 (0.95-1.731)	.104	1.174 (0.884-1.559)	.267	NA	NA	NA	NA
Tumor size (>5 *vs* ≤5)	1.420 (1.053-1.913)	.021	1.585 (1.195-2.102)	.001	1.264 (0.930-1.717)	.134	1.399 (1.049-1.864)	.022
Tumor number (multiple *vs* single)	1.719 (1.219-2.425)	.002	1.510 (1.090-2.092)	.013	1.638 (1.156-2.322)	.006	1.604 (1.147-2.244)	.006
Lymph node metastasis (positive *vs* negative)	2.183 (1.500-3.177)	<.001	2.419 (1.709-3.424)	<.001	1.839 (1.234-2.741)	.003	1.973 (1.364-2.853)	<.001
Microvascular invasion (positive *vs* negative)	1.294 (0.847-1.977)	.233	1.341 (0.899-2.002)	.150	NA	NA	NA	NA
Nerve invasion (positive *vs* negative)	1.906 (1.055-3.44)	.033	2.766 (1.669-4.582)	<.001	1.459 (0.795-2.677)	.222	2.358 (1.394-3.988)	.001
TILs^FOXP3^ (high *vs* low)	1.365 (1.016-1.835)	.039	1.383 (1.047-1.828)	.023	1.116 (0.819-1.521)	.487	1.116 (0.831-1.499)	.467
Tumor^CTLA-4^ (high *vs* low)	1.296 (0.963-1.745)	.088	1.125 (0.852-1.485)	.407	NA	NA	NA	NA
TILs^CTLA-4^ (high *vs* low)	1.393 (1.036-1.873)	.028	1.617 (1.222-2.141)	.001	NA	NA	NA	NA
Tumor^PD-L1^ (high *vs* low)	1.655 (1.219-2.247)	.001	1.394 (1.04-1.867)	.026	NA	NA	NA	NA
Tumor^PD-L1^/TILs^CTLA-4^ (G I/II/III *vs* G IV)	1.683 (1.210-2.341)	.002	1.806 (1.319-2.472)	<.001	1.566 (1.110-2.210)	.011	1.587 (1.141-2.206)	.006

Cox proportional hazards regression model. OS, overall survival; NA, not applicable; HBV, hepatitis B virus; TILsFOXP3, density of FOXP3+ TILs; TILsCTLA-4, density of CTLA-4+ TILs; TumorCTLA-4, expression level of CTLA-4+ tumor cells; TumorPD-L1, expression level of PD-L1+ tumor cells; G I, Patients with TumorPD-L1 High plus TILsCTLA-4 High; G II, Patients with TumorPD-L1 High plus TILsCTLA-4 Low; G III, Patients with TumorPD-L1 Low plus TILsCTLA-4 High; G IV, Patients with TumorPD-L1 Low plus TILsCTLA-4 Low; 95%CI, 95% confidence interval; HR, Hazard ratio.

### Distinct CTLA-4 Expression and Prognostic Role in ICC With Different Risk Factors

HBV/HCV infection and hepatolithiasis are risk factors for ICC (16). Our previous studies show that hepatolithiasis is an independent risk factor for ICC, and patients with hepatolithiasis had worse survival than patients with HBV infection or undefined risk factors (20). In the present study, we classified 290 patients with ICC into four subgroups according to HBV infection (HBV^+/-^) and hepatolithiasis (Stone^+/-^): 206 patients had HBV infection only (HBV^+^/Stone^-^), nine patients had hepatolithiasis only (HBV^-^/Stone^+^), seven patients had both hepatolithiasis and HBV infection (HBV^+^/Stone^+^), and 68 patients had undefined risk factors (HBV^-^/Stone^-^).

**Figure 4:**
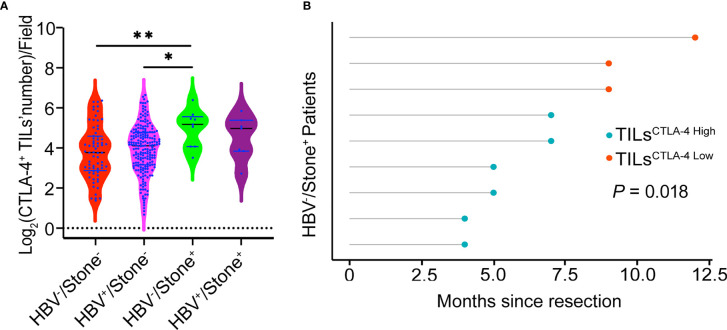
Relationship of the density of CTLA-4^+^ TILs to risk factors and prognosis. **(A)** Patients with HBV^-^/Stone^+^ ICC had a higher density of CTLA-4^+^ TILs in tumor samples compared with patients with HBV^+^/Stone^-^ ICC and HBV^-^/Stone^-^ ICC. **(B)** Patients with TILs^CTLA-4 High^ show a reduced OS compared with patients with TILs^CTLA-4 Low^ among nine patients with HBV^-^/Stone^+^ ICC. *P < 0.05 and **P < 0.01.

Our previous data also showed a PD1/PD-L1 signal in distinct expression mode and prognostic implication in different risk factor–related ICCs (20). Here, we further investigated CTLA-4 expression and prognostic significance in different risk factor–related ICCs. One-way ANOVA analysis showed that the density of CTLA-4^+^ TILs in tumor tissues from the four subgroups was significantly different (*P* = .031, **Figure 4A**). The density of TILs^CTLA-4^ in samples from patients with HBV^-^/Stone^+^ (37.4 ± 22.3/field) was higher than in patients with HBV^+^/Stone^-^ (21.2 ± 17.9/field, *P* = .013) and patients with HBV^-^/Stone^-^ (21.0 ± 20.3/field, *P* = .007). Interestingly, tumor samples from patients with HBV^-^/Stone^+^ showed a lower expression of PD-L1 than patients with HBV^+^/Stone^-^ as described in our previous study (20). We further investigated the prognostic influence of the density of TILs^CTLA-4^ in patients with HBV^-^/Stone^+^. The OS of the nine patients with HBV^-^/Stone^+^ was limited with a median of 7 months (from 4 to 12 months), and the TILs^CTLA-4 High^ patients with HBV^-^/Stone^+^ had poor survival (*P* = .018, **Figure 4B**).

## Discussion

We determined a profile of CTLA-4 expression with prognostic implication in a large cohort of patients with ICC. CTLA-4 was hyperactivated in tumor samples from patients with ICC, and the high density of CTLA-4^+^ TILs (TILs^CTLA-4 High^) was significantly correlated with malignant characteristics. Clinically, the density of CTLA-4^+^ TILs was an independent risk factor for OS in patients with ICC, and we found that patients with TILs^CTLA-4 High^ showed an unfavorable prognosis. These data indicate that CTLA-4 expression in TILs is an important factor for sustaining the inhibitory immune microenvironment in the clinical setting of ICCs. Thus, this provides a rationale for anti-CTLA-4 therapy in ICCs, at least in a subset of patients. Treg cells are considered the strongest inhibitor of antitumor activity, and CTLA-4 expression is essential for the activation of FOXP3^+^ T cells. Our data also show a positive relationship between the density of CTLA-4^+^ TILs and FOXP3^+^ TILs, which provides indirect evidence to support a role for CTLA-4 in the inhibitory immune microenvironment of ICCs. Furthermore, plenty of studies suggest that CTLA-4 on both activated conventional T cells and FoxP3+ Tregs is important for immunology suppression (33, 34), and it is demonstrated that Treg CTLA-4 blockade alone could not induce antitumor immunity, but it could augment the antitumor responses induced by CTLA-4 blockade of conventional T cells by using selective blockade of CTLA-4 on Treg or conventional T cell (35). Here, we show that both hyperactivated CTLA-4 and FOXP3 are related to an unfavorable prognosis, and the amount of CTLA-4+ TILs is higher than FOXP3+ Tregs, which indicates that besides FOXP3+ Tregs, other CTLA-4+ TILs may be involved in antitumor immune disorders. Thus, the overexpression of CTLA-4 reflects a more global immunomodulatory effect, not just Treg infiltration.

CTLA-4 maintains immune homeostasis through complex mechanisms; the cell-intrinsic model of CTLA-4 function describes that the cytoplasmic tail of CTLA-4 affects intracellular posttranslational modifications and regulates cellular localization of CTLA-4, and the cell-extrinsic model describes CTLA-4 acting through Tregs to exert its function (5). CD28 can costimulate T cell functions by affecting cytokine production, reducing the TCR signaling threshold for T cell activation and enhancing T cell proliferation and survival (36). CTLA-4 may act as an antagonist of CD28–ligand interaction by competing for ligand binding. Recent studies show that CTLA-4 expression was overactivated in several malignant tumors, such as melanoma and spinal chordoma (7, 37). Here, we also demonstrate that the CTLA-4 signal was activated in tumor tissues of ICCs. Patients with early recurrence of ICC had a higher density of CTLA-4 expression than patients without early recurrence. Moreover, the density of CTLA-4^+^ TILs is related to the density of FOXP3^+^ TILs. Therefore, CTLA-4 acts as a central element of immunologic tolerance to lessen the immune response in the tumor microenvironment (38). Further, the density of CTLA-4^+^ TILs in ICC tissues is related to aggressive clinicopathologic features, such as preoperative serum CA19-9, larger tumor size, lymph node metastasis, and high TNM stage. The augmentation of CTLA-4 expression in T cells could reduce the secretion of IFN-γ (39) and then facilitate malignant phenotypes, such as tumorigenesis and metastasis (40). Hence, CTLA-4^+^ TILs may be involved in the invasive behavior of ICC cells. Our data indicate that overexpression of CTLA-4 in TILs promotes the invasion and metastasis of ICC and may be a prognostic indicator in patients with ICC.

However, CTLA-4 expressed in tumor cells was not related to the prognosis of ICC. The role of CTLA-4 in tumor cells is controversial, and a previous study suggests that elevated CTLA-4 expression in tumor cells of NSCLC is predictive of a good outcome (41). CTLA-4 is constitutively expressed in a variety of tumor cell lines, such as breast, colon, kidney, lung, ovarian, and uterine cancers and in melanoma cell lines, and elevated CTLA-4 expression is associated with the induction of apoptosis through sequential activation of caspase-8 and caspase-3 (42), but the exact role and mechanism of CTLA-4 in ICC remain to be fully elucidated.

Furthermore, we determined a distinct expression profile of CTLA-4 and PD1/PD-L1 in ICC. PD-L1 was overexpressed in tumor cells, and CTLA-4 was activated in TILs but not tumor cells. CTLA-4 acts as an antagonist of CD28–ligand interactions by competing for ligand binding with CD80. Meanwhile, a large amount of CD80 expressed in antigen-presenting cells (APCs) directly competes with PD1 on the overlapping interface on PD-L1 to disrupt the combination of PD-L1/PD-1 and its inhibitory function in T cell activation. Further, PD-L1 inhibition reduces the expression of CD80 on APCs, and the effect could be offset by the blockage of CTLA-4. This molecular basis has implications for the combination of anti-PD-L1 and anti-CTLA-4 in treating ICC (43, 44). In the present study, patients with coactivation of PD1/PD-L1 and CTLA-4 signals presented the worst prognosis among patients with ICC. Moreover, the density of CTLA-4^+^ TILs was determined as an independent predictor of OS, and PD-L1 expression in tumor cells was an independent predictor of cumulative recurrence. Interestingly, combined CTLA-4^+^ TILs and PD-L1^+^ in tumor cells showed better sensitivity for predicting prognosis of ICCs in terms of OS and cumulative recurrence than that of overexpression of either CTLA-4 or PD-L1 alone. These data indicate that CTLA-4 is a good assistant of PD1/PD-L1 in the inhibitory TEM of ICC.

Moreover, we found that Tumor^PD-L1 High^ patients have more Tregs infiltrating into the ICC tumor than Tumor^PD-L1 Low^ patients. A previous study revealed that PD-L1 could promote Treg development and enhance Treg function (45), which provides implications in the synergistic use of anti-PD-L1 and anti-CTLA-4 therapies.

Additionally, distinct expression of CTLA-4 and PD1/PD-L1 was observed among different risk factors in ICC. Our data suggest that CTLA-4 overactivation in hepatolithiasis-related ICC is likely the predominant factor involved in sustaining the inhibitory immune environment, providing a promising therapeutic target for such patients.

In conclusion, our findings reveal elevated CTLA-4 and FOXP3 in ICC; the combined overexpression of CTLA-4 and PD-L1 is a good marker for predicting poor prognosis in ICCs and presents a potential target for ICI treatment strategies. These findings will be further evaluated in our clinical trial (**NCT**04634058) about the combination of anti-PD-L1 and anti-CTLA-4 in treating ICC patients, which is already in progress.

## Data Availability Statement

The raw data supporting the conclusions of this article will be made available by the authors, without undue reservation.

## Ethics Statement

The studies involving human participants were reviewed and approved by the Institutional Review Board of Zhongshan Hospital. Written informed consent for participation was not required for this study in accordance with the national legislation and the institutional requirements.

## Author Contributions

Concept and design: X-YH, G-MS. Data collection: L-XY, YC, X-JG, J-CL, H-YZ, Q-MS, G-HY, A-WK, Y-HShi, JZ, and JF. Experiments: X-JG, J-CL, H-YZ, Y-ZP, X-LM, P-FZ, and P-XH. Data analysis and visualization: X-JG, G-MS, X-YH, J-CL, Q-MS, G-HY, Y-HShi, J-BC, and RZ. Writing article: X-JG, X-YH, and G-MS. All authors contributed to the article and approved the submitted version.

## Funding

This study was supported by the National Key Research and Development Program of China (2019YFC1316000), the National Natural Science Foundation of China (81502028, 81972232, and 82072575), the Shanghai Municipal Natural Science Foundation (18410720700, 20JC1419103, and 21ZR1412200), the Clinical Research Plan of SHDC (SHDC2020CR1003A), and Sanming Project of Medicine in Shenzhen (No. SZSM202003009).

## Conflict of Interest

The authors declare that the research was conducted in the absence of any commercial or financial relationships that could be construed as a potential conflict of interest.

## Publisher’s Note

All claims expressed in this article are solely those of the authors and do not necessarily represent those of their affiliated organizations, or those of the publisher, the editors and the reviewers. Any product that may be evaluated in this article, or claim that may be made by its manufacturer, is not guaranteed or endorsed by the publisher.

## Supplementary Material

Supplementary Figure 1 Six-point scale for scoring CTAL-4 expression level on ICC tumor cells.

Supplementary Figure 2 Prognostic implications of CTLA-4 expression on ICC tumor cells or adjacent normal liver tissues. **(A, B)** Kaplan-Meier curves of OS and cumulative recurrence for patients with CTLA-4^high^ against patients with CTLA-4^low^, grouped by the density of CTLA-4+ lymphocytes in adjacent normal liver tissues. **(C, D)** Kaplan-Meier curves of OS and cumulative recurrence for patients with Tumor^CTLA-4 High^ against patients with Tumor^CTLA-4 Low^, grouped by expression of CTLA-4 in ICC tumor samples. "ns” refers to no significance.

Supplementary Figure 3 FOXP3 expression level in ICC patients with different characteristics. **(A)** Density of CTLA-4^+^ tumor infiltrating lymphocytes was higher than paired FOXP3^+^ infiltrating lymphocytes in the whole ICC cohort (*P* < 0.001, paired Student’s t-test). **(B)** Density of FOXP3^+^ tumor infiltrating lymphocytes was higher in Tumor^PD-L1 High^/TILs^CTLA-4 Low^ patients than Tumor^PD-L1 Low^/TILS^CTLA-4 High^ patients (*P* < 0.001, paired Student’s t-test). **(C)** Density of FOXP3^+^ tumor infiltrating lymphocytes was higher in Tumor^PD-L1 High^ patients than Tumor^PD-L1 Low^ patients (*P* < 0.001, paired Student’s t-test). ****P* < 0.001.

## Abbreviations

ICC, intrahepatic cholangiocarcinoma; HCC, hepatocellular carcinoma; ICIs, immune checkpoint inhibitors; CTLA-4, cytotoxic T-lymphocyte-associated antigen-4; FOXP3, forkhead box protein P3; PD1, programmed death 1; PD-L1, programmed death-ligand L1; TILs^CTLA-4 High^, high density of CTLA-4^+^ tumor-infiltrating lymphocytes; TILs^CTLA-4 Low^, low density of CTLA-4^+^ tumor-infiltrating lymphocytes; TILs^FOXP3 High^, high density of FOXP3^+^ tumor-infiltrating lymphocytes; TILs^FOXP3 Low^, low density of FOXP3^+^ tumor-infiltrating lymphocytes; Tumor^PD-L1 High^, high PD-L1 expression in tumors; Tumor^PD-L1 Low^, low PD-L1 expression in tumors; TMA, tissue microarray; TME, tumor microenvironment.
