# Supplementary figures and images for: CTLA-4 Synergizes With PD1/PD-L1 in the Inhibitory Tumor Microenvironment of Intrahepatic Cholangiocarcinoma
Source: Front Immunol. 2021 Aug 30;12:705378. doi: 10.3389/fimmu.2021.705378 (PMC8435712; doi:10.3389/fimmu.2021.705378)

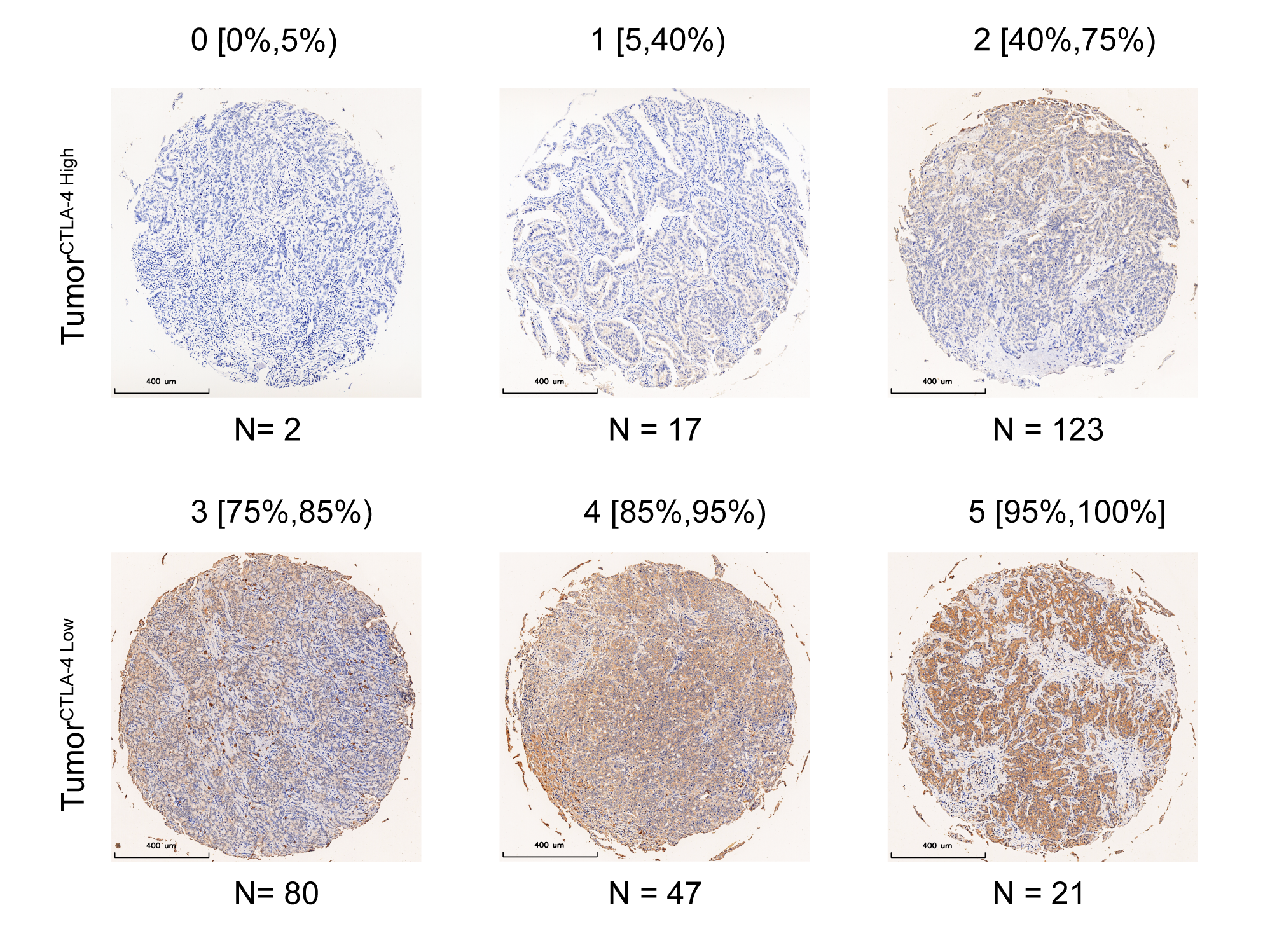

Supplement: Supplementary Figure 1 — Six-point scale for scoring CTAL-4 expression level on ICC tumor cells. [file Image_1.tif]

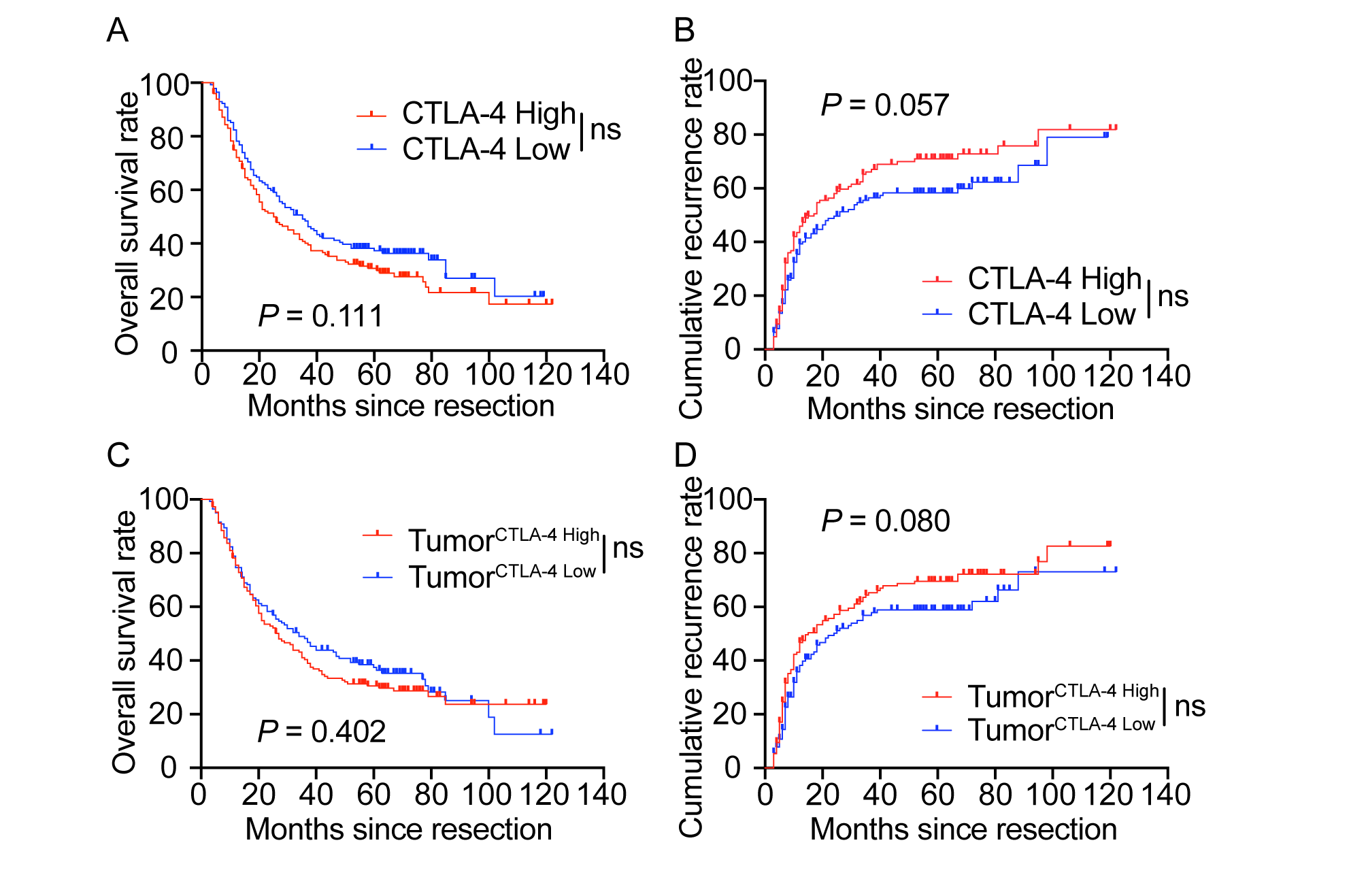

Supplement: Supplementary Figure 2 — Prognostic implications of CTLA-4 expression on ICC tumor cells or adjacent normal liver tissues. (A, B) Kaplan-Meier curves of OS and cumulative recurrence for patients with CTLA-4high against patients with CTLA-4low, grouped by the density of CTLA-4+ lymphocytes in adjacent normal liver tissues. (C, D) Kaplan-Meier curves of OS and cumulative recurrence for patients with TumorCTLA-4 High against patients with TumorCTLA-4 Low, grouped by expression of CTLA-4 in ICC tumor samples. "ns” refers to no significance. [file Image_2.tif]

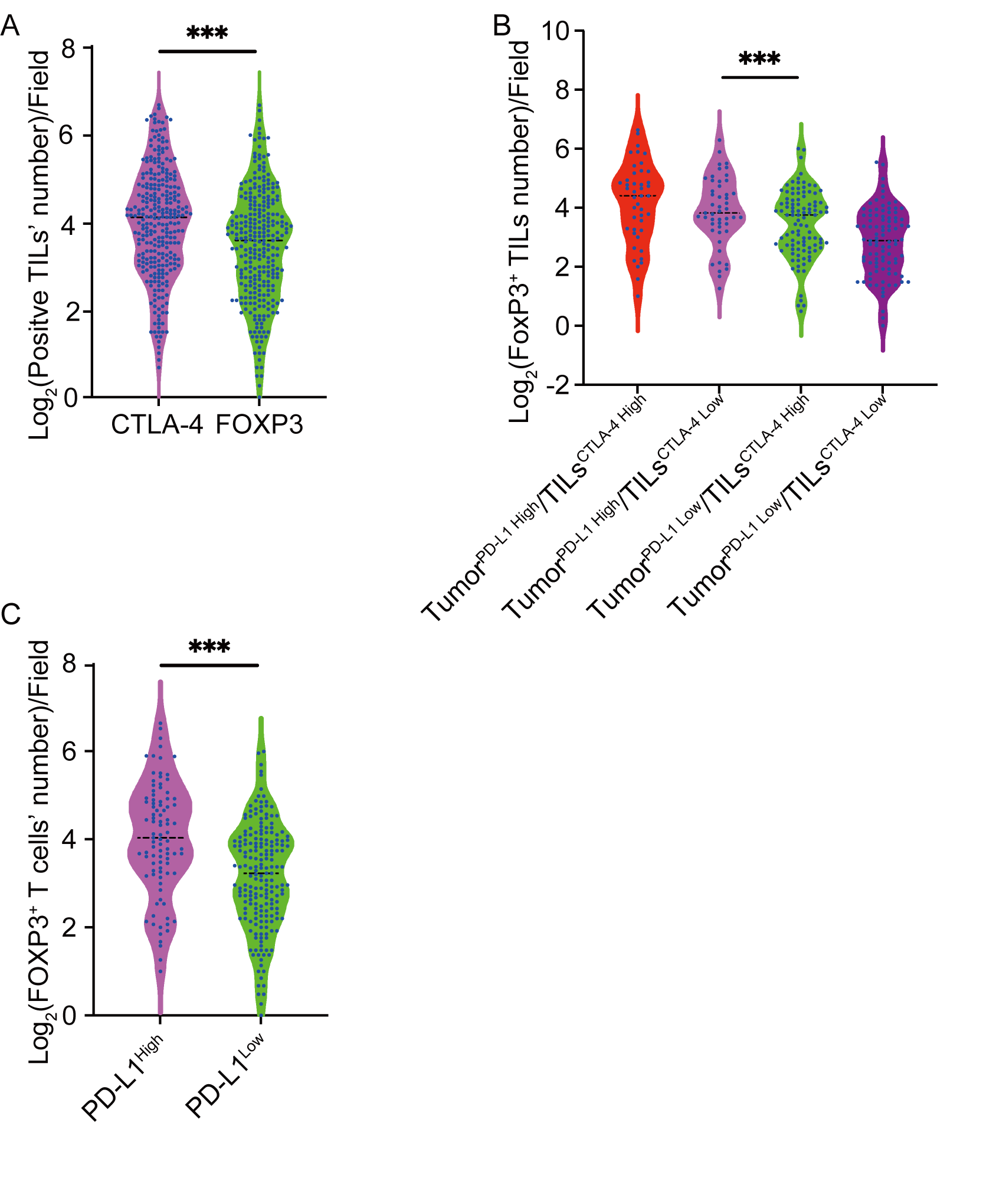

Supplement: Supplementary Figure 3 — FOXP3 expression level in ICC patients with different characteristics. (A) Density of CTLA-4+ tumor infiltrating lymphocytes was higher than paired FOXP3+ infiltrating lymphocytes in the whole ICC cohort (P < 0.001, paired Student’s t-test). (B) Density of FOXP3+ tumor infiltrating lymphocytes was higher in TumorPD-L1 High/TILsCTLA-4 Low patients than TumorPD-L1 Low/TILSCTLA-4 High patients (P < 0.001, paired Student’s t-test). (C) Density of FOXP3+ tumor infiltrating lymphocytes was higher in TumorPD-L1 High patients than TumorPD-L1 Low patients (P < 0.001, paired Student’s t-test). ***P < 0.001. [file Image_3.tif]
