# Supplementary material for: CTLA-4 Synergizes With PD1/PD-L1 in the Inhibitory Tumor Microenvironment of Intrahepatic Cholangiocarcinoma
Source: Front Immunol. 2021 Aug 30;12:705378. doi: 10.3389/fimmu.2021.705378 (PMC8435712; doi:10.3389/fimmu.2021.705378)
Supplement: Supplementary file 4 [file Table_1.docx]

**Supplementary Table 1. Univariate and multivariate analyses of characteristics associated with prognosis in 290 patients with ICC.**

| Characteristics | Univariate analysis | | | | Multivariate analysis | | | |
| --- | --- | --- | --- | --- | --- | --- | --- | --- |
|  | Cumulative recurrence | | OS | | Cumulative recurrence | | OS | |
|  | HR（95%CI） | *P value* | HR（95%CI） | *P value* | HR（95%CI） | *P value* | HR（95%CI） | *P value* |
| Age, years (>58 vs ≤58) | 0.823（0.612-1.106） | 0.196 | 0.894（0.677-1.180） | 0.429 | NA | NA | NA | NA |
| Sex (male vs female) | 1.174（0.865-1.594） | 0.302 | 1.187（0.890-1.583） | 0.243 | NA | NA | NA | NA |
| Hepatolithiasis (positive vs negative) | 1.923（0.979-3.777） | 0.058 | 3.932（2.303-6.713） | <0.001 | NA | NA | 4,200(2.398-7.356) | <0.001 |
| HBV infection (positive vs negative) | 1.000（0.715-1.399） | 0.999 | 0.885（0.649-1.205） | 0.436 | NA | NA | NA | NA |
| Liver cirrhosis (positive vs negative) | 1.173（0.839-1.640） | 0.350 | 1.224（0.896-1.670） | 0.204 | NA | NA | NA | NA |
| Tumor differentiation (III/IV vs I/II) | 1.283（0.950-1.731） | 0.104 | 1.174（0.884-1.559） | 0.267 | NA | NA | NA | NA |
| Tumor size (>5 vs ≤5) | 1.420（1.053-1.913） | 0.021 | 1.585（1.195-2.102） | 0.001 | 1.277(0.939-1.737) | 0.118 | 1.406(1.055-1.874) | 0.020 |
| Tumor number (multiple vs single) | 1.719（1.219-2.425） | 0.002 | 1.510（1.090-2.092） | 0.013 | 1.614(1.138-2.287) | 0.007 | 1.581(1.129-2.214) | 0.008 |
| Lymph node metastasis (positive vs negative) | 2.183(1.500-3.177) | <0.001 | 2.419(1.709-3.424) | <0.001 | 1.657(1.108-2.480) | 0.014 | 1.844(1.272-2.673) | 0.001 |
| Microvascular invasion (positive vs negative) | 1.294(0.847-1.977) | 0.233 | 1.341(0.899-2.002) | 0.150 | NA | NA | NA | NA |
| Nerve invasion (positive vs negative) | 1.906(1.055-3.444) | 0.033 | 2.766(1.669-4.582) | <0.001 | 1.539(0.840-2.819) | 0.163 | 2.525(1.501-4.246) | <0.001 |
| TILs^FOXP3^ (high vs low) | 1.365(1.016-1.835) | 0.039 | 1.383(1.047-1.828) | 0.023 | 1.092(0.799-1.492) | 0.582 | 1.166(0.866-1.569) | 0.311 |
| Tumor^CTLA-4^ (high vs low) | 1.296(0.963-1.745) | 0.088 | 1.125(0.852-1.485) | 0.407 | NA | NA | NA | NA |
| TILs^CTLA-4^ (high vs low) | 1.393（1.036-1.873） | 0.028 | 1.617(1.222-2.141) | 0.001 | 1.297(0.955-1.761） | 0.096 | 1.395(1.048-1.857) | 0.023 |
| Tumor^PD-L1^ (high vs low) | 1.655（1.219-2.247） | 0.001 | 1.394（1.040-1.867） | 0.026 | 1.548(1.124-2.133) | 0.008 | 1.130(0.827-1.546) | 0.442 |
| Tumor^PD-L1^/TILs^CTLA-4^ (G I/II/III vs G IV) | 1.683（1.210-2.341） | 0.002 | 1.806（1.319-2.472） | <0.001 | NA | NA | NA | NA |

Note: Cox proportional hazards regression model. Abbreviation: OS, overall survival; NA, not applicable; HBV, hepatitis B virus; TILs^FOXP3^, the density of FOXP3+ TILs; TILs^CTLA-4^, the density of CTLA-4+ TILs; Tumor^CTLA-4^, the expression level of CTLA-4+ tumor cells; Tumor^PD-L1^, the expression level of PD-L1+ tumor cells; G I, Patients with Tumor^PD-L1 High^ plus TILs^CTLA-4 High^; G II, Patients with Tumor^PD-L1 High^ plus TILs^CTLA-4 Low^; G III, Patients with Tumor^PD-L1 Low^ plus TILs^CTLA-4 High^; G IV, Patients with Tumor^PD-L1 Low^ plus TILs^CTLA-4 Low^; 95%CI, 95% confidence interval; HR, Hazard ratio.
